# *Calvia explanata* sp. n. (Coleoptera: Coccinellidae: Coccinellinae) from the Indian Subcontinent

**DOI:** 10.3897/BDJ.2.e1164

**Published:** 2014-08-25

**Authors:** J. Poorani

**Affiliations:** † National Bureau of Agriculturally Important Insects, P.B. No. 2491, H.A. Farm Post, Bellary Road, Hebbal, Bangalore 560024, India

**Keywords:** *Calvia explanata*, new species, Coccinellidae, Indian Subcontinent

## Abstract

*Calvia explanata* sp. n. (Coleoptera: Coccinellidae), externally similar to and commonly misidentified as *Calvia albida* Bielawski, is described from India and Nepal.

## Introduction

The genus *Calvia* Mulsant (1846) is distributed throughout the Palaearctic and Oriental regions and contains over 20 species. A single species, *Calvia quatuordecimguttata* (L.) occurs widely in the northern half of North America (Booth 1997). Booth (1997) reviewed the species of the genus from the Indian Subcontinent, most of them being endemic to the Himalayas, and provided a key to species along with biological notes. Accounts of some of these species with illustrations are also given by Yu (2010) and Ren et al. (2009). A species of *Calvia* externally very simillar to *Calvia albida* Bielawski (1972) and labelled as the latter was found in collections from northeastern India and Nepal and is described and illustrated here.

## Materials and methods

For preparation of male and female genitalia, whole specimens were immersed in warm soapy water for 10 minutes. The abdomen was detached gently with a minuten pin and kept overnight in 10% KOH. The genitalia were dissected in distilled water and transferred to glycerol for studies and imaging. After examination, the genitalia were transferred to microvials and pinned beneath the respective specimens. The following measurements were made using the measurement module of a Leica M205A stereo microscope: total length, from apical margin of clypeus to apex of elytra (TL); total width, across both elytra at their widest point (TW = EW); pronotal length, from the middle of anterior margin to the base of pronotum (PL); pronotal width at its widest (PW); elytral length along suture from apex to base including scutellum (EL). Images of whole specimens and their diagnostic characters were taken using a Leica DFC 420 camera attached to a Leica M205A stereo microscope. Composite images were generated from image stacks using Combine ZP and touched up in Adobe Photoshop Elements 11. The specimens studied are deposited in the following collections: NBAII – National Bureau of Agriculturally Important Insects, Bangalore; NPC – National Pusa Collection, Indian Agricultural Research Institute, New Delhi.

## Taxon treatments

### Calvia explanata sp. n.

urn:lsid:zoobank.org:act:E5D6455D-C0EF-4164-81F8-C8CE013B6AD5

#### Materials

- **Type status:** Holotype. **Occurrence:** recordedBy: Hemchandra; individualCount: 1; sex: Male; **Location:** country: India; stateProvince: Sikkim; verbatimLocality: Pantok; **Event:** eventDate: 2008-05-21; habitat: on *Alnus* sp.; **Record Level:** institutionCode: NBAII
- **Type status:** Paratype. **Occurrence:** recordedBy: P.P. Bhattacharjee; sex: Female; **Location:** country: India; stateProvince: Sikkim; verbatimLocality: Mangam; **Event:** eventDate: 2013-03-13; **Record Level:** institutionCode: NBAII
- **Type status:** Paratype. **Occurrence:** recordedBy: Hemchandra; individualCount: 2; **Location:** country: India; stateProvince: Sikkim; verbatimLocality: Pantok; **Event:** eventDate: 2008-05-21; habitat: on *Alnus* sp.; **Record Level:** institutionCode: NBAII
- **Type status:** Paratype. **Occurrence:** recordedBy: Bhakta, B.; individualCount: 1; sex: Male; **Location:** country: India; stateProvince: West Bengal; verbatimLocality: Darjeeling Dt: Bom Busty; **Event:** eventDate: 1990-III-26/27
- **Type status:** Paratype. **Occurrence:** recordedBy: W. Wittmer; C. Baroni; individualCount: 1; **Location:** country: Nepal; stateProvince: Godavari; **Event:** eventDate: 1976-05-25
- **Type status:** Paratype. **Occurrence:** recordedBy: J. Schneider; individualCount: 1; sex: Female; **Location:** country: Nepal; stateProvince: Bagmati prov.; verbatimLocality: Nagarjun Forest; verbatimElevation: 1387m; verbatimLatitude: 27.45N; verbatimLongitude: 85.17E; **Event:** eventID: Nepal Expedition Jan Farkac, David Kial, & Jan Schneider, 2000; samplingProtocol: at light
- **Type status:** Paratype. **Occurrence:** sex: Male; **Location:** country: India; stateProvince: Sikkim; verbatimLocality: Mangam; **Event:** eventDate: 2013-03-08; **Record Level:** institutionCode: NBAII

#### Description

TL: 7.50–9.00 mm; TW: 6.00–8.00 mm; TL/TW: 1.07–1.50; PL/PW: 0.45–0.48; EL/EW: 0.88–1.03. Male: Form (Fig. 1a, b, c) broadly oval, dorsum convex, broadest a little before middle of elytra, elytra nearly as wide as long; glabrous except head with silvery white hairs. Dorsum bright lemon yellow to yellowish green except lateral margins of pronotum and elytra transparent, pronotum with an indistinct M-shaped marking. Antennae, mouthparts and ventral side yellowish brown; ventral surface covered with short, silvery white pubescence. Head with clypeal margin truncate between lateral projections; eyes prominent, inner ocular margins anteriorly distinctly divergent. Antenna 11-segmented, elongate, >2× as long as interocular distance, with a moderately long 3-segmented club, terminal antennomere oval, apically flattened. Punctures on head shallow, separated by 2–5 diameters, interspaces between punctures with distinct, reticulate microsculpture. Pronotum finely punctate, punctures separated by 2–5 diameters, interspaces with strong, reticulate microsculpture on disc, more obsolete towards lateral margins. Elytral punctures slightly larger and more widely spaced than those on pronotum, separated by 3–6 diameters, interspaces shinier than that on pronotum, with microsculpture; lateral expansions of elytra with larger and coarser punctures. Prosternal intercoxal process convex, without carinae. Mesoventrite medially semi-circularly emarginate. Metaventrite with discrimen. Epipleuron wide, deeply concave, distinctly descending externally. Meso- and metatibiae with a pair of apical spurs. Tarsal claws appendiculate. Abdominal postcoxal line (Fig. 2a) very short, not reaching posterior margin of ventrite 1. Posterior margin of ventrite 5 shallowly and widely emarginate, that of ventrite 6 medially more deeply emarginate. Male genitalia (Fig. 2a, b, c, d, e) as illustrated, tegmen in lateral view (Fig. 2b) with parameres longer than penis guide, apices covered with dense, elongate hairs; penis guide in inner view (Fig. 2c) elongate, cylindrical in anterior half, posterior half distinctly narrowed, triangular with a tubular apex; penis (Fig. 2d, e) with a distinct capsule, strongly arched, apically produced into a strongly curved process (Fig. 2e).

Female: Externally similar to male. Female genitalia (Fig. 2f) as shown, spermatheca bulky, sperm duct somewhat abruptly thickened a little after basal third; infundibulum present, composed of a pair of apparently lamellate structures.

#### Diagnosis

This species is externally similar to *Calvia albida*, *Calvia flaveola* Booth and *Calvia championorum* Booth. It is particularly close to *Calvia albida* (Fig. 1d) in having a mottled elytral pattern with several smaller spots. Both *Calvia albida* and *Calvia explanata* share a similar distribution range in India and are found in northeastern India and Nepal. *Calvia albida* is also distributed in China (Ren et al. 2009; Yu 2010). *Calvia explanata* differs from *Calvia albida* in having a distinctly wider, more circular body outline and explanate elytral margins. The male genitalia are also distinctive with the penis guide narrower and more elongate and the penis with an elongate, membranous apical process. In *Calvia albida*, the penis guide is distinctly broader up to a little beyond middle and the posterior half is much less narrowed and the penis has a more robust basal capsule and the apex is different. The male genitalia of *Calvia albida* were illustrated by Bielawski (1972) and Ren et al. (2009). The female genitalia in *Calvia albida* (Fig. 3) has a distinctly bulkier spermatheca (Fig. 3b) with a long, more or less uniformly thick sperm duct and the infundibulum is differently structured.

#### Etymology

The specific epithet is an adjective of Latin origin and refers to the explanate elytral margins.

#### Distribution

India (Sikkim; West Bengal). Nepal.

#### Biology

This species seems to have an arboreal habitat like many other species of *Calvia* (Booth 1997) as some of the specimens examined were collected on *Alnus* sp. (label data).

#### Taxon discussion

*Calvia* is not a very well defined genus with some members having aberrant characters. This species is placed in *Calvia* by the following combination of characters as per the diagnosis given by Gordon (1985): anterolateral angles of clypeus produced forward, lateral margins of pronotum and elytra explanate, middle and hind tibial apices with a pair of spurs each, abdominal postcoxal line incomplete, and tarsal claws appendiculate. Gordon (1985) in his diagnosis of *Calvia* mentioned that female genitalia lack infundibulum, but female genitalia of many Oriental species of *Calvia*, including *Calvia explanata*, have a distinct infundibulum.

## Supplementary Material

XML Treatment for Calvia explanata

## Figures and Tables

**Figure 1a.**
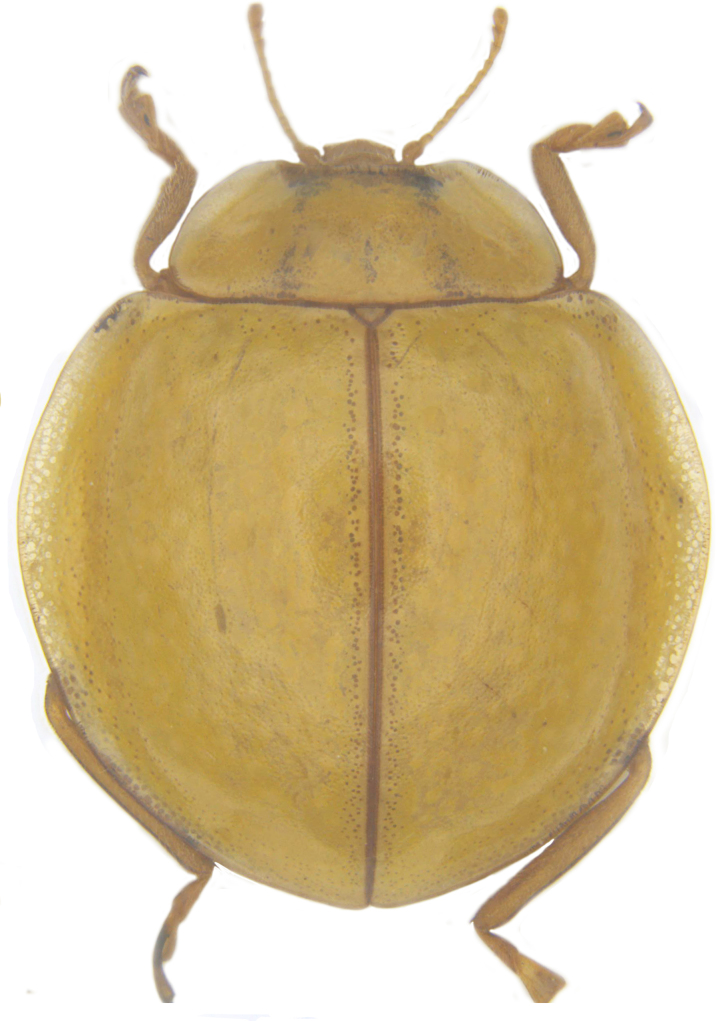
*Calvia explanata* sp. n.: Dorsal habitus.

**Figure 1b.**
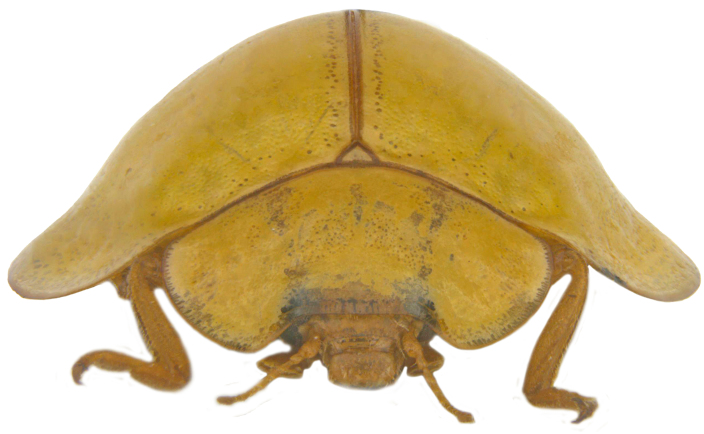
*Calvia explanata* sp. n.: Frontal view.

**Figure 1c.**
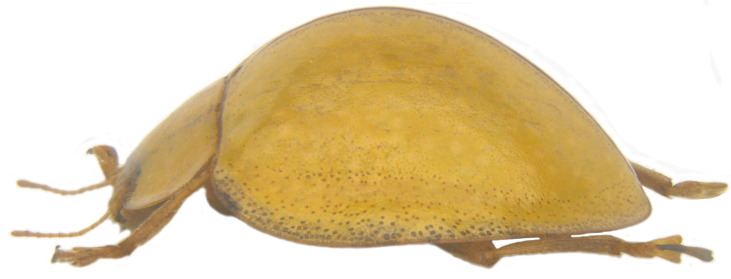
*Calvia explanata* sp. n.: Lateral view.

**Figure 1d.**
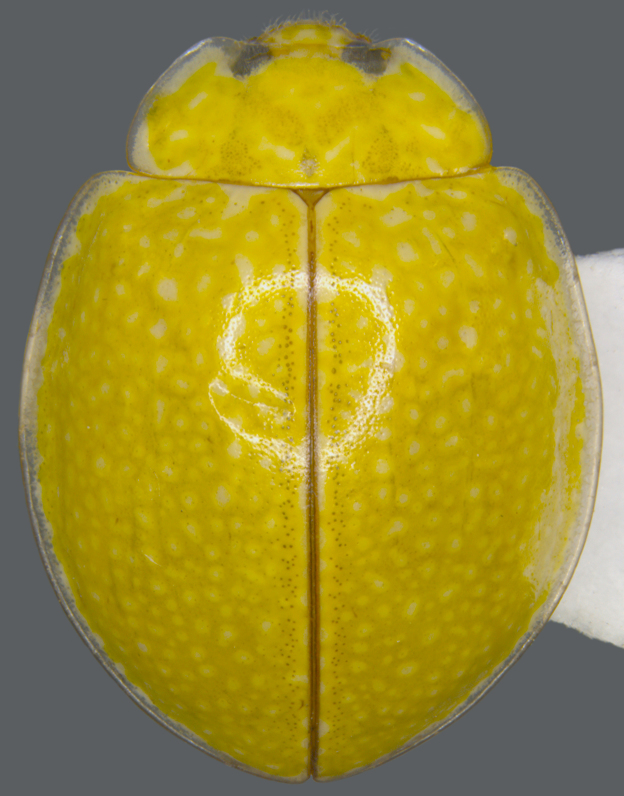
*Calvia albida*: Dorsal habitus.

**Figure 2a.**
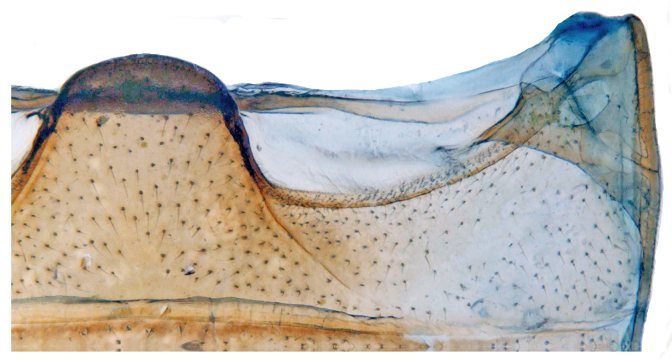
Abdominal postcoxal line.

**Figure 2b.**
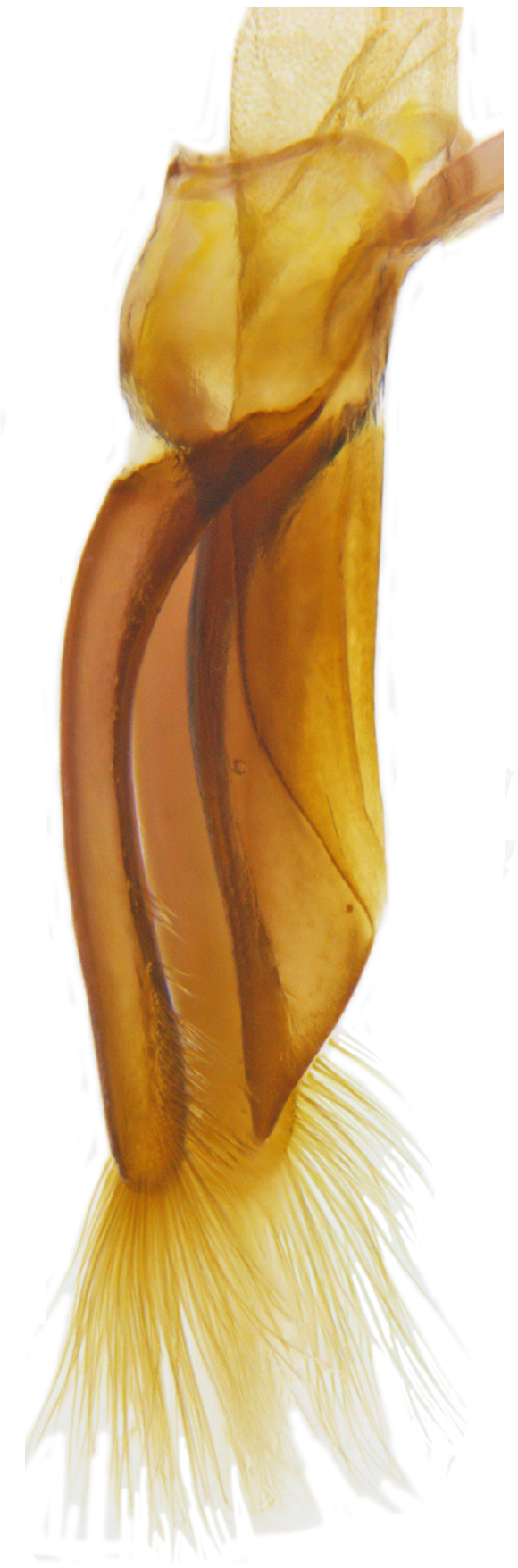
Male genitalia: Tegmen, lateral view.

**Figure 2c.**
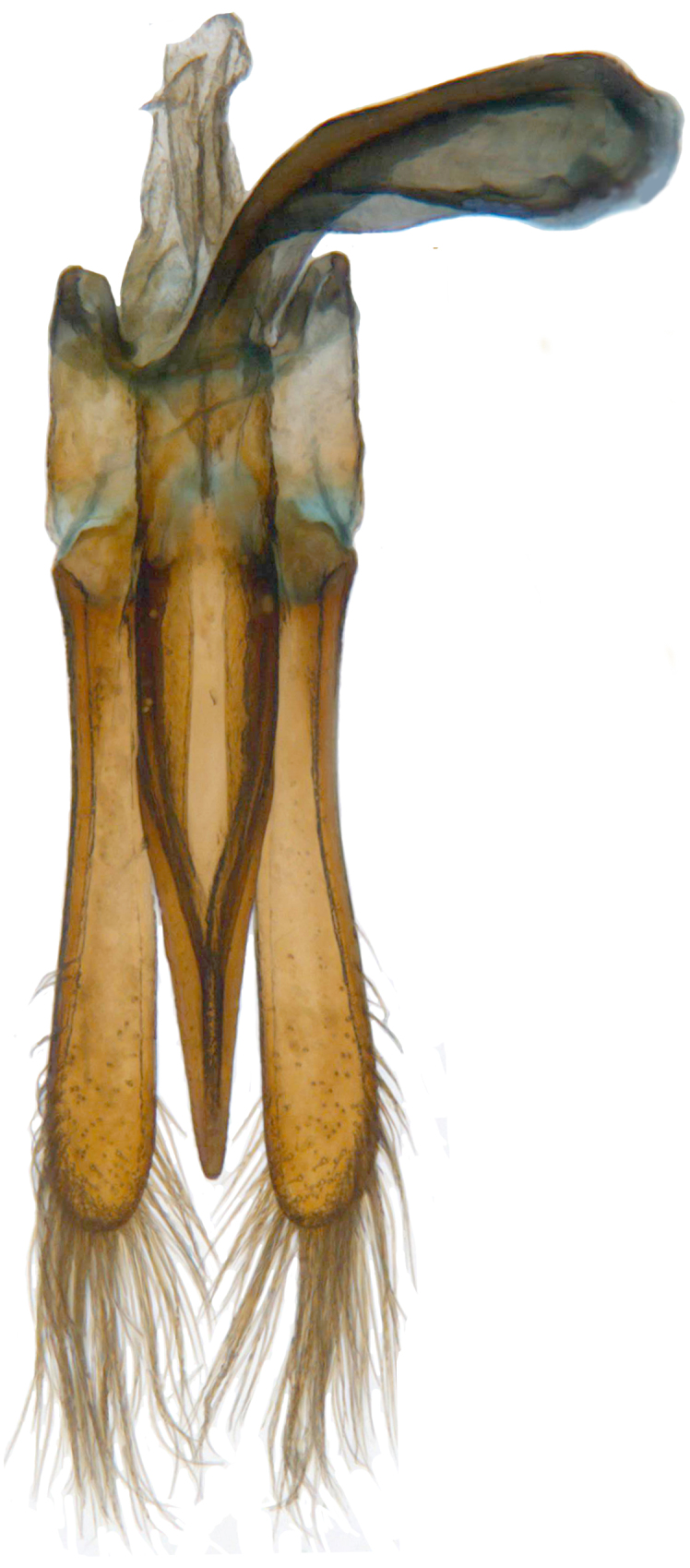
Male genitalia: Tegmen, inner view.

**Figure 2d.**
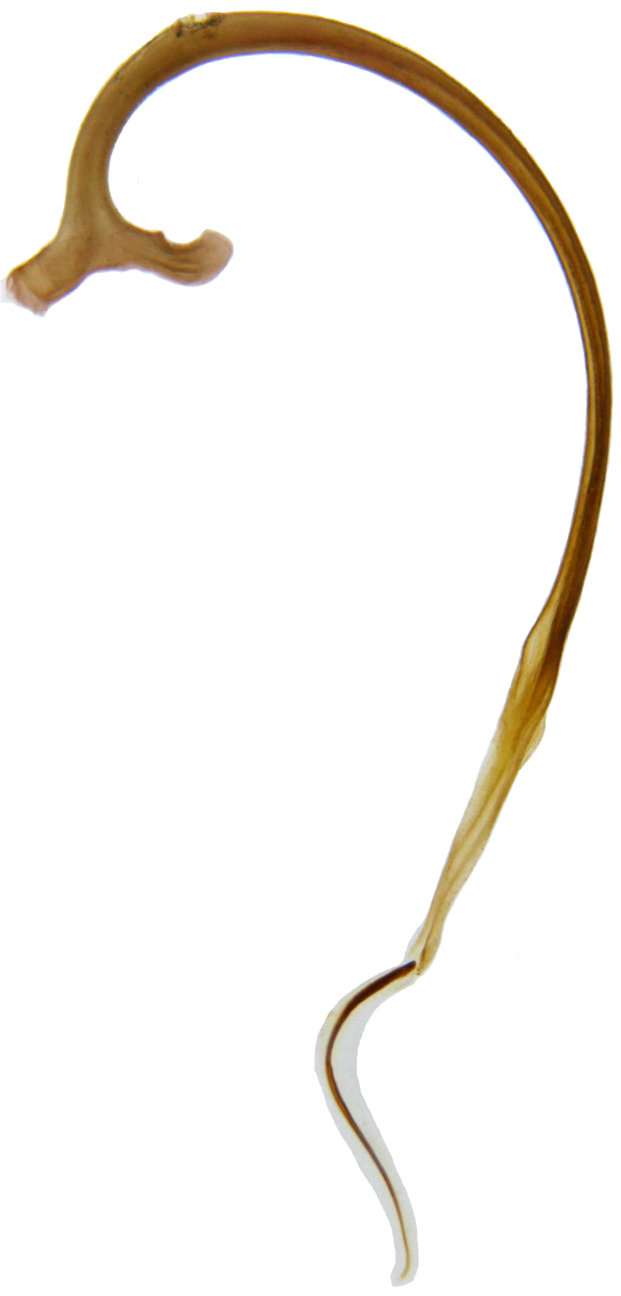
Male genitalia: Penis.

**Figure 2e.**
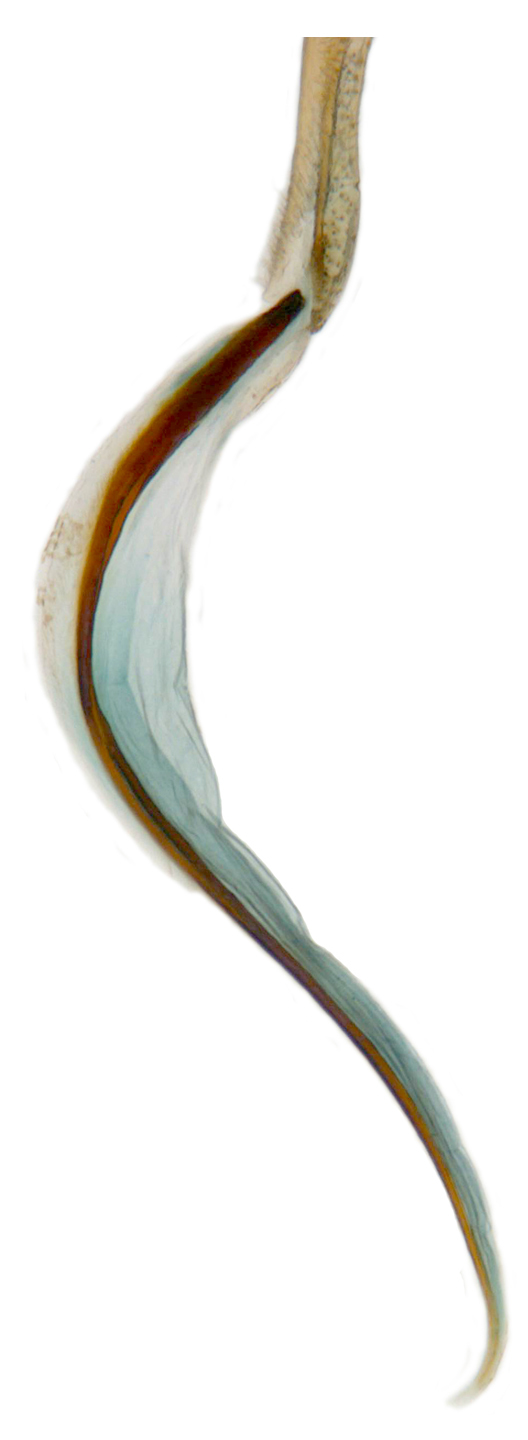
Male genitalia: Penis apex, magnified.

**Figure 2f.**
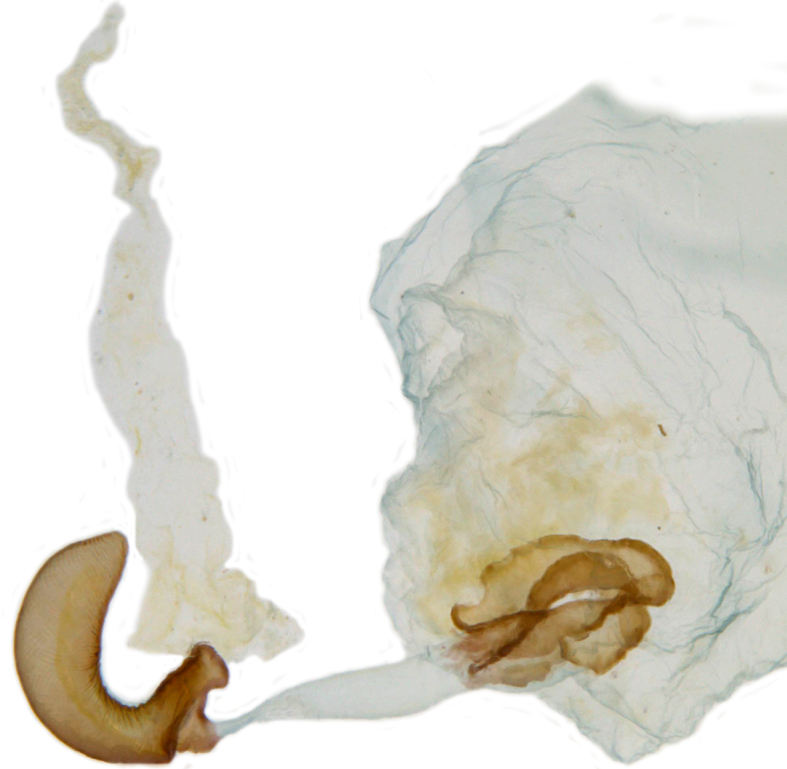
Female genitalia, spermatheca with accessory gland, sperm duct and distal part of bursa.

**Figure 3a.**
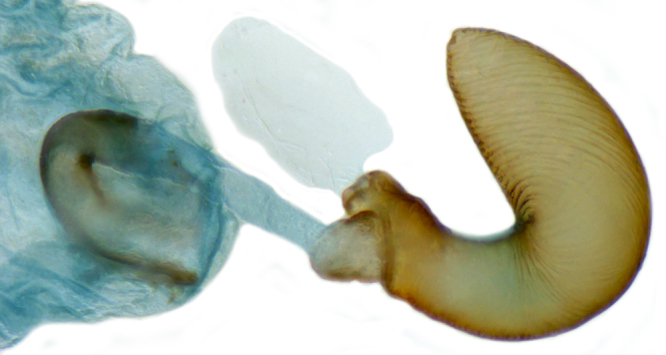
Female genitalia.

**Figure 3b.**
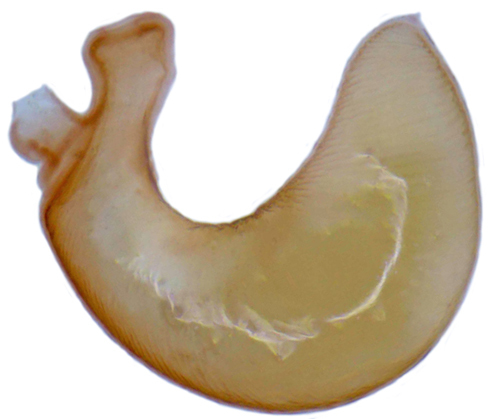
Spermatheca.
